# Parietotemporal Stimulation Affects Acquisition of Novel Grapheme-Phoneme Mappings in Adult Readers

**DOI:** 10.3389/fnhum.2018.00109

**Published:** 2018-03-23

**Authors:** Jessica W. Younger, James R. Booth

**Affiliations:** ^1^Department of Communication Sciences and Disorders, University of Texas at Austin, Austin, TX, United States; ^2^Department of Psychology and Human Development, Vanderbilt University, Nashville, TN, United States

**Keywords:** transcranial direct current stimulation, parietotemporal cortex, reading acquisition, artificial orthography, reading skill

## Abstract

Neuroimaging work from developmental and reading intervention research has suggested a cause of reading failure may be lack of engagement of parietotemporal cortex during initial acquisition of grapheme-phoneme (letter-sound) mappings. Parietotemporal activation increases following grapheme-phoneme learning and successful reading intervention. Further, stimulation of parietotemporal cortex improves reading skill in lower ability adults. However, it is unclear whether these improvements following stimulation are due to enhanced grapheme-phoneme mapping abilities. To test this hypothesis, we used transcranial direct current stimulation (tDCS) to manipulate parietotemporal function in adult readers as they learned a novel artificial orthography with new grapheme-phoneme mappings. Participants received real or sham stimulation to the left inferior parietal lobe (L IPL) for 20 min before training. They received explicit training over the course of 3 days on 10 novel words each day. Learning of the artificial orthography was assessed at a pre-training baseline session, the end of each of the three training sessions, an immediate post-training session and a delayed post-training session about 4 weeks after training. Stimulation interacted with baseline reading skill to affect learning of trained words and transfer to untrained words. Lower skill readers showed better acquisition, whereas higher skill readers showed worse acquisition, when training was paired with real stimulation, as compared to readers who received sham stimulation. However, readers of all skill levels showed better maintenance of trained material following parietotemporal stimulation, indicating a differential effect of stimulation on initial learning and consolidation. Overall, these results indicate that parietotemporal stimulation can enhance learning of new grapheme-phoneme relationships in readers with lower reading skill. Yet, while parietotemporal function is critical to new learning, its role in continued reading improvement likely changes as readers progress in skill.

## Introduction

Reading is a fundamental educational skill important for academic and vocational success (Gerber, 2012), yet not every child develops into a fluid reader. Poor readers exhibit a number of behavioral deficits related to reading including phonological awareness, grapheme-phoneme (letter-sound) mapping and reading fluency (Shaywitz and Shaywitz, 2005; Siegel, 2006). These behaviors have been related to a primarily left hemisphere network of brain regions that show reduced activation in individuals with poor reading ability, including inferior frontal, parietotemporal and occipitotemporal areas (Richlan et al., 2011; Richlan, 2014). While neuroimaging research has converged on neural patterns associated with poor reading, which neural patterns are causes compared to consequences of poor reading is not yet clear. One proposed theory of failed reading is that reduced activity in parietotemporal regions involved in grapheme-phoneme integration results in impaired learning of letter-sound mappings critical for reading (Pugh et al., 2001; Schlaggar and McCandliss, 2007; Blau et al., 2010; Blomert, 2011). This idea is supported by developmental research showing the most consistently underactivated region in children with dyslexia is parietotemporal cortex, though adults with dyslexia show greater reduced activation in occipitotemporal areas associated with orthographic processing (Richlan et al., 2011).

Further support is provided by studies of pre-readers with and without risk for dyslexia as well as intervention studies. The most consistently reported anatomical and functional differences reported are in parietotemporal areas (Vandermosten et al., 2016). Indeed, successful reading intervention is marked by increases in parietotemporal regions in both children (Simos et al., 2002, 2007; Shaywitz et al., 2003, 2004; Odegard et al., 2008; Yamada et al., 2011) and adults (Eden et al., 2004). Though intervention modulates activity in inferior frontal and occipitotemporal areas, activity in parietotemporal cortex has also shown to be predictive of the response to intervention (Odegard et al., 2008; Rezaie et al., 2011a,b), further supporting its potential causal role in development of reading skill. However, reduced function in occipitotemporal activation has also been observed (Specht et al., 2009; Raschle et al., 2011, 2012; Vandermosten et al., 2016). Without longitudinal data from sufficiently large samples to determine whether at risk children do go on to develop dyslexia, the neural differences associated with risk for dyslexia that are also significant predictors of the development of dyslexia cannot be established.

Thus, while parietotemporal activity and an understanding of grapheme-phoneme mappings have been established as critical for reading improvement for struggling readers, whether parietotemporal activity is causally related to learning grapheme-phoneme mappings is not yet clear. To better support remedial reading programs for both children and adults, we must better understand the role of parietotemporal activity in new learning. The neural effects associated with learning of new grapheme-phoneme relationships in non-impaired individuals can be examined in adults by training them to read a new orthography. This new orthography could be a previously unknown writing system or an artificial one created to control for or manipulate various factors such as mapping consistency or visual complexity of characters. Similar to intervention and developmental studies, orthographic learning studies in adults have shown learning related increases in left hemisphere reading regions (Hashimoto and Sakai, 2004; Bitan et al., 2005; Callan et al., 2005; Mei et al., 2014; Takashima et al., 2014; Taylor et al., 2017). Further, parietotemporal areas do show training related increases specifically related to accuracy gains on grapheme-phoneme mappings in the new language (Hashimoto and Sakai, 2004; Callan et al., 2005; Takashima et al., 2014). Parietotemporal cortex is also involved in reading untrained “transfer” words in the newly learned script that is similar to the activity found during reading pseudowords in English (Mei et al., 2014; Takashima et al., 2014; Taylor et al., 2017). However, relationships between individual differences in activation and learning have largely been left unexamined, and the predictive relationship between parietotemporal activity and learning outcomes seen in intervention studies has not yet been established in orthographic learning studies. One study has demonstrated a positive relationship between increases in parietotemporal activity and increases in accuracy to trained words, but there was no relationship with transfer word or retention performance (Deng et al., 2008). However, this study focused on training semantic-orthographic, not grapheme-phoneme, relationships. The only studies examining pre-training neural predictors of orthography learning in adults have been restricted to orthographic processing areas in occipitotemporal regions (Xue et al., 2006; Cao et al., 2013). Even less is known about the neural predictors of long-term retention of the newly learned orthography, though one study indicates visual attention prior to learning may be an important factor (Cao et al., 2013). Thus, whether there is a relationship between parietotemporal region activation and learning of grapheme-phoneme mappings, including the ability to transfer and retain this information, is yet unknown.

One method used to experimentally examine the role of parietotemporal regions in reading is neuromodulation. Neuromodulation affects neural activity in the affected region(s) which often leads to physiological or behavioral changes (Nitsche et al., 2008; Nitsche and Paulus, 2011; Stagg and Nitsche, 2011; Horvath et al., 2015, 2016). One such neuromodulation tool is transcranial direct current stimulation (tDCS), in which a low electrical current is delivered to the scalp. Anodal (positive) current is thought to reduce the firing threshold of neurons in brain regions under the electrode, while cathodal (negative) current is thought to raise the firing threshold (Nitsche and Paulus, 2000; Stagg and Nitsche, 2011). Anodal stimulation is therefore presumed to enhance behavior, whereas cathodal stimulation inhibits it (Jacobson et al., 2012) though this traditional pattern does not always hold true (Wiethoff et al., 2014; Bestmann et al., 2015). Studies applying anodal tDCS to parietotemporal areas have demonstrated stimulation-related improvements in reading ability in low-skill adults (Turkeltaub et al., 2012; Younger et al., 2016) and adolescents with dyslexia (Costanzo et al., 2016a). These studies thus support previous research showing a relationship between changes in parietotemporal function and changes in reading skill. However, this relationship has yet to be extended to new learning.

Only one previous study has paired stimulation with reading skill training to determine whether stimulation to parietotemporal areas can facilitate reading intervention in children with dyslexia (Costanzo et al., 2016b). Children received training on reading speed and grapheme-phoneme mappings with real or sham stimulation aimed at enhancing left lateralization of parietotemporal cortex. Reading speed was trained via tachistoscopic presentation of words in which words were flashed on the screen for a limited time range after which children were to read the word aloud. Grapheme-phoneme mappings were trained via tasks in which children had to correctly complete the written form of a word that corresponded to a presented picture and a task in which children rearranged syllables to form real words. Children who received real stimulation during training showed improved accuracy for low-frequency words and improved reading speed for nonwords compared to children who received training without stimulation. Gains in performance for the stimulation group compared to the sham group were also maintained for a 6-week period, showing stimulation can have lasting impact on performance. Further, because the effects were found on skills not directly related to the training received, there is some support for the effects of stimulation transferring to untrained skills. This study provides a demonstration of the potential for parietotemporal stimulation to enhance reading interventions. Yet, because both reading speed and grapheme-phoneme mappings were trained, it is unclear whether stimulation benefitted both or only one process. Further, stimulation only affected two of eight measures of reading, and while there is some evidence of transfer, that there was no effect on the behaviors more directly related to the training is at odds with previous tDCS research. Thus, while stimulation to parietotemporal areas do seem to affect reading related learning, many open questions remain.

The goal of the current study, therefore, was to examine the effect of parietotemporal stimulation on learning new grapheme-phoneme mappings in adults varying in their reading skill. We taught adults to read a novel writing system for English, allowing us to examine learning rates when only the visual representation of a word is novel, not the sound or meaning. This design ensured any potential effects could be attributed to learning new visual representations and not due to potential influences of processing novel or meaningless sounds. We then compared learning curves between readers who received real or sham stimulation to the parietotemporal cortex. We predicted individual differences in reading skill prior to learning would interact with stimulation to affect learning. Specifically, we expected that stimulation would increase learning curves for low skill readers more than high skill readers because of diminishing returns on the effect of stimulation. To ensure that grapheme-phoneme rules were learned and readers did not simply memorize mappings of entire word forms, we examined performance on both trained and novel, untrained transfer words. Similar effects of stimulation on performance across trained and untrained transfer words would indicate parietotemporal stimulation affected acquisition of these new grapheme-phoneme mappings. Finally, we also determined whether parietotemporal stimulation facilitated long-term maintenance of newly learned material, which would indicate lasting benefits of stimulation facilitated learning, as seen in previous studies.

## Materials and Methods

### Participants

In total, 89 right-handed 18–35-year-old native English speaking adults with normal or corrected-to-normal vision enrolled in the study. All participants reported no history of neurological disorder, psychiatric disorder, significant head trauma, hearing loss, substance abuse, seizure or migraine, metal implants and current pregnancy. Of the initial 89, 79 participants completed all training sessions and were considered for the analysis. An additional 16, eight in each stimulation group, were excluded for showing no evidence of learning during training (performance significantly above chance at both the final training session and final test of words). The remaining 63 participants included in the analysis had at least average (>85 standard score) intelligence as measured by the Wechsler Abbreviated Scale of Intelligence (WASI; Wechsler, 2008). All participants scored within two standard deviations above or below the mean on all standardized assessments (>70 and <130 standard score), with the exception of the WASI, in which the maximum score was 135. Participants were pseudorandomly assigned to receive real or sham stimulation to the left inferior parietal lobe (L IPL) based on standardized testing performance at baseline to ensure equivalent performances and number of participants across stimulation groups. Of those who met all performance criteria, 32 (25 female) received real stimulation to the L IPL and 31 (21 female) received sham stimulation. Two-sample *t*-tests revealed no significant effects of group on all group characteristics and standardized test performance as reported in Table 1.

**Table 1:** IQ was measured by the Performance subscale of WASI.

	Age	IQ	Word identification	Word attack	Sight word efficiency	Pseudoword decoding efficiency	Reading rate
*Stimulation (n = 31)*	23 (5)	114 (9)	106 (7)	101 (9)	105 (9)	97 (9)	2.25 (0.29)
							Range 1.70–3.00
*Sham (n = 32)*	24 (4)	115 (9)	107 (6)	101 (9)	103 (11)	97 (9)	2.17 (0.33)
							Range 1.22–2.68

*Groups were similar in demographics and performed similarly on all measures of reading skill. IQ was measured by the Performance sub-scale IQ index from the Wechsler’s Abbreviated Scale of Intelligence. Note: all tests have μ = 100, σ = 15, except for Reading Rate*.

### Procedure

**Figure 1:**
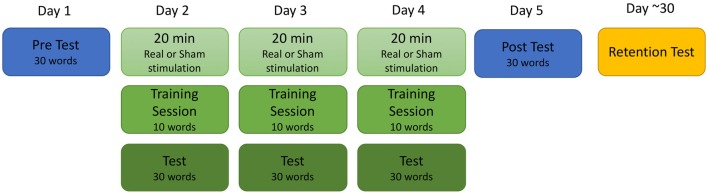
Depiction of the training procedures.

Participants took part in single-blind sham controlled study completed over a total of six sessions. The training procedure is depicted in Figure 1. The first five sessions occurred between 24 h and 48 h of each other, and the sixth took place approximately 4 weeks after the completion of the fifth session (mean 4.7 weeks; range 1.4–8 weeks). During the first session, participants completed both a battery of standardized tests to determine reading ability and a baseline test of the training stimuli. During the second, third and fourth days, participants received 20 min of real or sham stimulation followed by training on 10 new words in the artificial orthography. Finally, they were tested on the entire training set of 30 words and a unique set of 20 untrained “transfer” words that followed the same grapheme-phoneme pattern as the trained words. On the fifth day, participants did not receive stimulation but completed a cumulative test of all 30 trained words and 20 unique transfer words to assess final knowledge of the artificial orthography. The sixth retention test session was similar to the fifth session; participants did not receive stimulation and were tested of all 30-trained words as well as a test of 20 unique untrained transfer words.

#### Standardized Testing

Participants completed a battery of standardized tests to assess general intelligence and reading ability. Intelligence was measured by the nonverbal-scale IQ index from the Performance sub-scale WASI (Wechsler, 1999). All participants had at least average intelligence (>85 standard score), per inclusionary criteria. Reading fluency was assessed by the Phonemic Decoding Efficiency (PDE) and Sight Word Efficiency (SWE) subscales of the Test of Word Reading Efficiency (TOWRE; Torgesen et al., 1999). The TOWRE requires participants to read as many pseudowords (PDE) or words (SWE) as possible in 45 s. Untimed reading skill was assessed by the Woodcock Johnson Test of Achievement III Word Identification and Word Attack subtests (Woodcock et al., 2007). These tests require participants to read increasingly difficult words (Word Identification) or pseudowords (Word Attack) with no time requirements. The TOWRE tests were additionally administered on the fifth day after completion of the final testing session to assess whether stimulation may have affected English reading ability.

In keeping with previous studies demonstrating an effect of tDCS on reading skill, we used SWE performance as the metric of reading skill for analytical purposes. However, the maximum standard score an adult can earn on the SWE is 113 (mean 100, SD 15). Because we wanted to assess a wide-range of reading abilities (two standard deviations above or below the mean), we used a modified metric. In addition to recording the total number of words participants correctly read in 45 s in accordance with the standardized protocol, we allowed all participants to read the entire list of words and recorded the time to read the list in its entirety. We then calculated a reading rate score by dividing the total number of correctly read words by the number of seconds required to complete the list. To relate reading rate to standardized test performance, we calculated the reading rate that would correspond to standard scores of 87, 100 and 113. The corresponding reading rates were 1.911, 2.177 and 2.28 words per second, respectively.

#### Artificial Orthography and Training Procedure

Participants were trained on an artificial orthography using a Klingon-like script created for a previous successful artificial orthography training study (Brennan and Booth, 2015). The orthography is composed of letter-like characters that correspond to English phonemes and are combined to make English words. By learning real English words instead of pseudowords, participants had access to semantic representations during learning. This design approximates learning an orthography for which the linguistic sounds and their meaning are known. The artificial orthography was previously pruned for symbols resembling English letters. The remaining graphemes were randomly assigned to correspond with 10 consonant (/b/, /d/, /g/, /k/, /m/, /n/, /p/, /r, /s/, /t/) and five vowel (/ӕ/, /i/, /ɪ/, /ɑ/, /ə/) phonemes. Words were constructed using a CVC structure with a transparent 1:1 grapheme to phoneme ratio such that each letter represents one and only one sound. This design means that though participants learned English words, the training words may not have had the same number of letters as their English counterparts. For example, “beet” is written with three graphemes, corresponding to the /b/, /i/ and /t/ phonemes present in the word. The low grapheme-phoneme ratio was used to encourage a decoding-based learning strategy and discourage a holistic strategy of memorizing whole symbols or attempting to translate the symbols into English. Further, it maximized the potential for transfer to new words. Inconsistencies between number of consonant graphemes in English and artificial orthography words occurred in 25% of the 130 words participants were exposed to throughout the course of training and testing. Of those, inconsistencies primarily related to digraphs, e.g., the digraph “ck” was represented with one letter, “k”, in the artificial orthography. Only seven words had inconsistent consonant spellings not related to digraphs (e.g., “cat” was represented as “kat”). For a full list of stimuli, see Supplementary Table S1.

Each participant learned a total set of 30 words, broken in to three training lists of 10 words each. In each training list, each consonant was used twice: once as the first and once as the last letter of a word. Each vowel was used twice. Five sets of 20 “transfer” words were also created following the same procedures. These transfer sets were tested but not trained, allowing us to determine how well participants generalized the underlying grapheme-phoneme rules present in the orthography. Sets of words were equated for English word frequency, and the construction of word lists ensured that the occurrence of each letter was equated. As such, while semantics was accessible to participants, it could not have affected learning. That is, words could not be predicted based on information from the first two letters alone, and all three letters needed to be processed to correctly identify the word.

**Figure 2:**
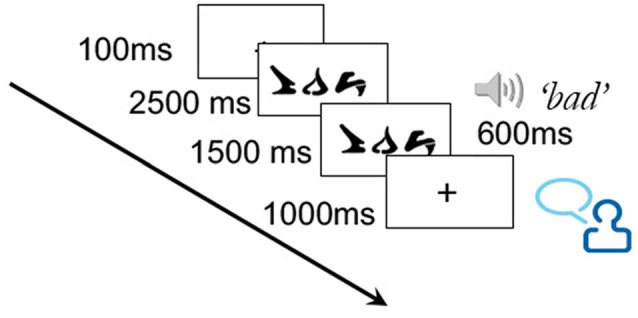
Illustration of training trial.

Training took place over the course of three sessions, during which 10 of the 30 words from the training set were each presented twice. This low number of training trials per word was to minimize potential ceiling effects on learning. On each training trial, a word was presented for a total of 4000 ms. After 2500 ms, the correct corresponding auditory word was played, which lasted approximately 600 ms. The word remained on the screen for an additional 1500 ms following the pronunciation. Participants were instructed to say the correct word aloud at some point during the trial (see Figure 2). While the verbal responses were not recorded, the requirement to say the word ensured attention to the task and aided in the learning process.

After each training block, the entire set of 30 training words as well as one set of transfer words were tested. As such, the number of words participants were explicitly trained on prior to testing differed each training day. In the first and second training sessions, 20/30 and 10/30 words respectively were similar to transfer words in that the correct pronunciation of the symbol was not known, however, participants had been previously exposed to these symbols during the baseline test. During each test trial, one word was presented on the screen for 2000 ms followed by an auditory word. Participants were asked to determine whether the presented stimuli are from the same word (i.e., if the auditory and visual items match), and press a button in response. Participants were not provided feedback on these tests. Each visual word was presented twice: once matched with its correct audio, and once mismatched. The foil for a target word was a word from the set that shares at least one letter with the target word. In order to prevent learning from the test, foil pairs were always presented together. A different transfer set was tested after each training block to ensure transfer words were completely novel for each test. Each test thus consisted of 60 trials of trained words and 40 trials of untrained transfer words.

#### tDCS

Direct current was administered using a battery-driven DC stimulator device (NeuroConn) via two saline-soaked electrodes (5 cm × 5 cm; 25 cm^2^). The anode electrode was placed over the L IPL (P3) according to the international 10-20 system for electroencephalography (EEG) electrode placement (Herwig et al., 2003). The cathode (return) electrode was placed over the contralateral supraorbital frontal region. During real stimulation, 1.5 mA of current (current density 0.06 mA/cm^2^) was delivered for 20 min. During sham stimulation, the machine ramped up to 1.5 mA for 30 s, then extinguished over a 5 s fade-out. Using this procedure allows participants to feel the initial sensations (e.g., tingling or itching) associated with stimulation without any after-effects of stimulation being induced (Nitsche and Paulus, 2000). These stimulation parameters replicate the parameters used previous reading studies (Turkeltaub et al., 2012; Younger et al., 2016) and are within the safety limits established in prior studies on humans and animals (Iyer et al., 2005; Nitsche et al., 2008; Bikson et al., 2009). All participants watched a silent movie for 20 min during the actual or sham stimulation (Antal et al., 2007; Gill et al., 2015).

### Analysis

Accuracy to trained and transfer words across the six testing sessions were analyzed using a multivariate latent growth curve modeling approach (McArdle and Nesselroade, 2003) using Mplus v7.3 (Muthén and Muthén, 2012). Data were analyzed using full information maximum likelihood (FIML) estimate to take all data, including participants with missing data, into account. Latent growth curve modeling estimates an intercept, the starting value for a measurement, and a slope to represent the intercept’s change across all measurement points. Accuracy during the baseline testing session was entered as the initial measurement or intercept (path weight of 0) for both trained and transfer words. The slope therefore estimated the amount of accuracy change beyond the baseline session that occurred over the remaining sessions relative to 0, for all participants, regardless of initial baseline performance. Because the shape of the learning curve may not be linear, path weights for the three training sessions were allowed to be freely estimated while the path weight for the testing session (day 5) was fixed to 4. Since no additional training with the artificial orthography occurred between the final testing session and the retention testing session, the path weight for the retention session was also fixed to 4. Further, we expected a direction change to occur between the first 5 days and the retention test such that accuracy would increase over the first five sessions but decrease at the retention test. Therefore, we entered an additional slope to model the change between the final testing session and the retention test. For these second intercepts and slopes, all testing sessions were fixed to 0 with the retention test session fixed at 1. This approach allowed us to examine effects of stimulation and skill on both acquisition of the new orthography and its retention separately. Model fit was assessed using the root mean squared error of approximation (RMSEA) and the Comparative Fit Index (CFI). CFI compares fit of the target model to a null model in which it is assumed all variables are uncorrelated. CFI scores range between 0 and 1, with 1 indicating the best fit. RMSEA is an absolute measure of fit that indicates the difference between the observed and predicted covariance matrix with values ranging from 0 to 1, and 0 indicating a perfect fit on the target model. Traditionally, a CFI > 0.90 and RMSEA < 0.05 is considered good model fit. CFI values between 0.80 and 0.90 and RMSEA values between 0.05 and 0.08 are generally considered acceptable but suboptimal (Hooper et al., 2008).

#### Covariates

To determine the effect of variables on intercept and slopes, intercept and slopes were regressed on covariates entered into the model. Covariates of interest were stimulation group, reading skill, and interaction between reading skill and group. Group was entered as a dummy coded variable with as 2 representing real stimulation and 4 sham. Reading rates centered around the rate corresponding to the mean standard test score of 100 (2.177) were entered to represent reading skill. A group by skill interaction term was determined by multiplying the group dummy code variable by the centered reading rate and entered as an interaction term. Additional covariates were entered to control for previously demonstrated effects of age, IQ and sex on stimulation. Age was centered around 18, the youngest age in the sample, IQ was centered on the population mean standard score (100), and sex was dummy coded as 1 or 2. These values were then each multiplied by the group dummy variable to obtain an interaction term for each. The intercept and slopes were additionally regressed on the three interaction terms.

#### Missing Data

Not all participants had usable data from all testing sessions. Seventeen participants (nine stimulation, seven sham group) did not complete the retention test session. In some cases, individual responses were not recorded due to technical errors or slow response time. Trials were excluded if the response time was less than 300 ms or no response was recorded (including responses that did not correspond to the instructed keyboard response). Data from a testing session was considered unusable and entered into the latent growth curve model as missing if the number of missing responses was greater than statistically different from chance (22 and 13 missing responses for trained and transfer tests respectively). Thus, in all included time points, participants responded to at least 63% (trained) and 67% (transfer) of all trials, whether correct or incorrect. All participants had at least three time points of useable data and missingness was not systematically related to reading skill or stimulation group. The number of participants for each time point ranged from 45 (the retention test) to the full set of 63 participants. All time points met minimum covariance coverage (10%) with values ranging from 68.3% to 100%.

## Results

Standardized parameter estimates of each covariate on the intercept and training and transfer slopes are reported in Table 2. Standardized parameters indicate the estimated standard deviation change in intercept and slopes given one standard deviation change in the predictor variable.

**Table 2:** Parameter estimates (standard error) for each covariate on the intercept, training slope and retention slope for trained and transfer words.

		Trained words		Transfer words	
		Estimate	(SE)	Estimate	(SE)
*Intercept*	Stimulation group	0.193	(0.631)	−0.133	(0.613)
	Skill	2.756*	(0.802)	2.481*	(0.874)
	Group by skill	−2.635*	(0.835)	−2.391*	(0.888)
	Group by age	0.098	(0.436)	0.294	(0.415)
	Group by IQ	−0.489	(0.420)	−0.541	(0.394)
	Group by sex	−0.060	(0.275)	0.030	(0.267)
*Training slope*	Stimulation group	−0.181	(0.239)	0.005	(0.244)
	Skill	−1.148*	(0.462)	−1.358*	(0.450)
	Group by skill	1.269*	(0.452)	1.347*	(0.448)
	Group by age	−0.424*	(0.160)	−0.268	(0.166)
	Group by IQ	0.491*	(0.156)	0.372*	(0.163)
	Group by sex	0.130	(0.103)	0.030	(0.267)
*Retention slope*	Stimulation group	−0.838*	(0.285)	−0.74	(0.439)
	Skill	−0.820	(0.623)	1.984*	(0.838)
	Group by skill	0.672	(0.641)	−1.282	(0.957)
	Group by age	0.949*	(0.173)	0.274	(0.386)
	Group by IQ	−0.235	(0.261)	−0.068	(0.387)
	Group by sex	0.001	(0.144)	0.300	(0.212)

Positive effect of group indicates an advantage for sham, and a negative effect of group indicates an advantage for stimulation. Skill had a significant effect and interaction with group on training slopes for both trained and transfer words. Stimulation affected the retention slope for trained words while skill affected the retention slope of transfer words. *p < 0.05.

### Trained Words

Model fit indices indicate the model fit the data for trained words well (RMSEA = 0.039; CFI = 0.978). Significant effects of skill and group by skill interaction term on the intercept indicate higher skilled readers tended to perform better at baseline. However, lower skill readers tended to show the lowest performance at baseline within the stimulation group while higher skill readers tended to have the lowest performance within the sham group.

**Figure 3:**
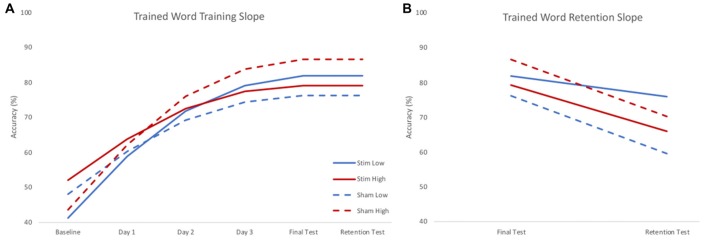
Model estimated training **(A)** and retention **(B)** slopes for trained words. During training **(A)**, low skill readers (blue) benefitted from real stimulation (solid), showing steeper learning curves compared to those who received sham stimulation (dashed). High skill readers (red), showed less training related gains following stimulation (solid) compared to those who received sham stimulation (dashed). During retention **(B)**, those who received real stimulation (solid) showed less forgetting compared to those who received sham stimulation (dashed). Plots reflect the model estimated performance for an 18-year-old male with average intelligence (reflecting mean centered scores of 0) at two standard deviations below (low) and above (high) group mean reading skill.

There were significant effects of skill and group by skill interaction on the training slope after controlling for significant effects of interactions between stimulation group and age and IQ. A negative parameter estimate for skill indicates the training slope became smaller as skill increased. Because skill was treated as a continuous variable, we used the model to estimate the effect of group on the training slope at three skill levels to interpret the interaction effects. The three skill levels chosen were the centered mean and two standard deviations above or below the centered mean reading rate calculated using the mean and standard deviation of the centered reading rate in the sample (mean 0.111; SD 0.309). There was a significant positive effect of group at the lower skill level, but a significant negative effect of group at the higher skill level. Thus, stimulation benefited the training slope for lower skill readers, but stunted the training slope for higher skill readers. Given the significant effects of variables of no interest (such as the interaction between stimulation group and age), results were visualized by calculating the model estimated performance of the same participant across different levels of stimulation group and skill. In this way, the visualization of results shows the effect of stimulation group and skill in the absence of any effects of demographic variables. Figure 3A shows the model predicted performance for an 18-year-old male with average IQ (reflecting a mean score of 0 for these covariates of no interest) and either two standard deviations below (low skill) or above (high skill) the mean centered reading rate of the sample. All subsequent plots use these same parameters. It should be noted that despite differences in intercept (baseline performance) the effects of slope are calculated assuming an intercept of 0. As such, slope would only be affected by baseline performance if participants reached a ceiling for accuracy, preventing further possible improvements. As Figure 3 shows, participants did not reach ceiling; indeed, the group with the highest baseline accuracy achieved only the third highest accuracy at the final testing session.

There was no effect of skill on retention slope, rather, there was a significant effect of stimulation group after controlling for a significant group by age interaction. The sham group showed a steeper negative retention slope compared to the stimulation group. Thus, regardless of skill level, the stimulation group forgot less in the interval between the training and the retention test (see Figure 3B).

### Transfer Words

Model fit indices indicate the model did not fit the data for transfer words as well as trained words (RMSEA = 0.095; CFI = 0.787). Given work showing model fit indices tend to over-reject acceptable models in samples <100 (Kenny et al., 2015), the model was considered acceptable. The same pattern of results was found for the intercept of the training slope for transfer words with higher skill readers tending to have higher baseline performance with the interaction showing the same pattern of results within each group.

**Figure 4:**
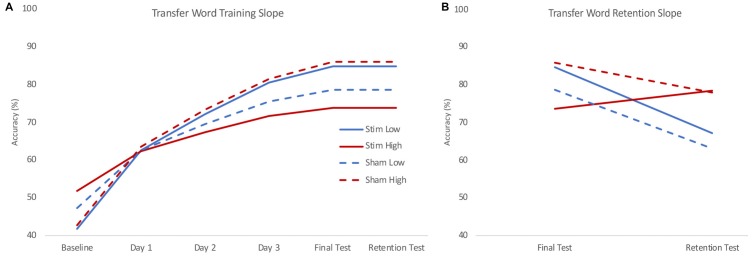
Model estimated training **(A)** and retention **(B)** slopes for transfer words. During training **(A)**, low skill readers (blue) who received real stimulation (solid) showed steeper learning curves for transfer to novel words compared to those who received sham stimulation (dashed). High skill readers (red) were less able to transfer letter knowledge to newly learned words following stimulation (solid) compared to those who received sham stimulation (dashed). During retention **(B)**, high skill readers regardless of stimulation group (red) showed less decline in transfer compared to low skill readers (blue) who show a decrease in transfer. Plots reflect the model estimated performance for an 18-year-old male with average intelligence (reflecting mean centered scores of 0) at two standard deviations below (low) and above (high) group mean reading skill.

The training slope for transfer words also showed similar effects of skill and group by skill interaction, though there was only an additional significant effect of group by IQ interaction, not group by age as in the trained words data. Skill again had a negative effect on the training slope for transfer words. We performed the same simple slope calculations to determine the direction of effect in the group by skill interaction employed for the trained words. We obtained a similar pattern of results, with stimulation tending to benefit the training slope at lower levels of reading skill and stunting it for higher levels of reading skill (see Figure 4A). However, in this case, the effect of group at the lower reading skill level was not significant (see Table 3).

**Table 3:** Parameter estimates (standard error) for the effect of group for lower, average and higher skill readers.

	Trained words		Transfer words	
	Estimate	(SE)	Estimate	(SE)
*Lower skill*	−1.869*	(0.857)	−1.668	(0.938)
*Average skill*	−0.092	(0.535)	0.381	(0.588)
*Higher skill*	1.685*	(0.869)	2.431*	(0.953)

Positive effect of group indicates an advantage for sham, and a negative effect of group indicates an advantage for stimulation. Stimulation improved the training curve for low skill readers, but interfered with learning for high skill readers. *p < 0.05.

On the retention slope, there was a significant effect of skill but not group, contrasting the results of the trained word model. Reading skill had a positive effect on the retention slope for transfer words, indicating poorer readers showed a greater decrease in performance on transfer words between the final training session and the retention test (see Figure 4B). However, stimulation had no effect on retention, nor did it interact with skill to significantly affect retention.

## Discussion

The goal of this study was to determine whether parietotemporal stimulation could improve learning and long-term retention of new grapheme-phoneme relationships in lower reading skill adults. As predicted, parietotemporal stimulation improved acquisition rates for lower skilled adults. Yet, parietotemporal stimulation negatively impacted higher skill adults’ learning curves. The effects of stimulation also transferred to untrained material, with stimulation benefitting transfer word learning curves of lower skill readers and impairing that of higher skill readers. Further, stimulation improved long-term retention of trained material across all skill levels. This study supports prior research showing pre-learning parietotemporal activity predicts response to reading intervention and goes beyond previous orthographic learning studies that have shown training affects parietotemporal cortex activity by suggesting that parietotemporal activity can affect new learning, including transfer and long-term retention.

That stimulation affected individuals of varying skill levels differently suggests our readers did have variation in the composition of their reading network at baseline, most likely in the parietotemporal area targeted by stimulation. By manipulating parietotemporal function, we provide evidence to support the importance of this region for word learning from explicit instruction (Wong et al., 2007; Richardson et al., 2010; López-Barroso et al., 2013). The results of the current study suggest that for adult learners, new learning depends on an optimal balance between semantic, phonological and orthographic information. Connectionist models of reading suggest semantics is reached via two pathways, an orthography to semantics pathway and an orthography to phonology to semantics pathway. These pathways both contribute to word reading, but the division of labor between the two differs depending on the type of word being read (e.g., exception words, high frequency words, pseudowords; Harm and Seidenberg, 1999, 2004; Seidenberg, 2005). The phonologically mediated pathway is less efficient, but initially dominant when learning to read, whereas the more efficient orthography to semantics pathway is formed and strengthened over time. Even when the more efficient orthography to semantics pathway is fully formed, the phonologically mediated pathway remains a significant contributor to word reading, with the sum of outputs from the two pathways being greater than the output of the either pathway on its own (Harm and Seidenberg, 2004). According to this model, one reason for lower reading skill may be a weaker phonologically mediated pathway. Lower skill but non-impaired readers, such as the readers in the current study, may still achieve reasonable reading skill by relying more on the orthography to semantics pathway. The orthography to semantics pathway thus plays a dominant role regardless of word type, which ultimately results in an overall less efficient reading network (Harm and Seidenberg, 2004). In our study, parietotemporal stimulation likely strengthened this phonologically mediated pathway, resulting in better learning in lower skill readers. However, this same increase to an already strong phonologically mediated pathway in higher skilled readers may have caused this less efficient pathway to be a stronger contributor throughout the course of learning which prevented the more efficient orthography to semantics pathway from effectively contributing as it developed later in learning. Indeed, neural connectivity studies in typical adult readers have suggested that readers who tend to rely on one processing stream regardless of word type are more likely to have lower reading ability compared to those readers whose neural strategy shifts depending on word type (Levy et al., 2009). Thus, readers who continued to rely on the phonologically mediated pathway could successfully acquire the orthography, but at a slower rate than those readers who were able to successfully shift the division of labor between the two pathways over the course of learning.

Stimulation had a positive effect on learning grapheme-phoneme relationships, but only for readers who showed initial lower reading skill, as measured by real word reading fluency. These findings underscore the importance of considering baseline performance when determining the effect of stimulation, and may reconcile conflicting results amongst reports of the effect of stimulation on reading in healthy adults (Turkeltaub et al., 2012; Thomson et al., 2015; Younger et al., 2016; Westwood and Romani, 2017). Studies examining either lower skill adults or adults with dyslexia have demonstrated positive effects of left hemisphere stimulation on reading ability (Turkeltaub et al., 2012; Younger et al., 2016). However, two studies have found a null effect on reading ability after left hemisphere stimulation, with one showing a positive effect after right hemisphere stimulation (Thomson et al., 2015; Westwood and Romani, 2017). These two studies, however, studied adults within the typical range of reading ability and do not account for individual differences in baseline performance. As suggested by previous research, the effects of stimulation may have been reduced when examining all skill levels together, resulting in a null effect (Benwell et al., 2015; Hsu et al., 2016).

The differential effect of stimulation depending on baseline skill level is consistent with previous stimulation studies as well (Tseng et al., 2012; Benwell et al., 2015; Hsu et al., 2016; Katz et al., 2017), yet the results of our study extend these studies in an important way. Previous research has indicated potential diminishing returns of stimulation, with the benefit of stimulation decreasing as baseline performance increases (Tseng et al., 2012; Katz et al., 2017). Our study shows not just diminishing returns but a significant negative effect of stimulation as skill increases. While other studies have shown anodal stimulation generally thought to have a positive effect on behavior can in some cases have a negative effect (Antal et al., 2007; Jacobson et al., 2012; Sandrini et al., 2012), to our knowledge, ours is the first study showing anodal stimulation can have a positive effect for some individuals, and a negative effect for others, depending on baseline skill level (though see Wiethoff et al., 2014 for other examples of individual differences in direction of effect). This result supports our previous findings reported in Younger et al. (2016) in which we demonstrated stimulation can have a negative and not just a null effect.

The effects of parietotemporal stimulation extended beyond explicitly trained words to novel transfer words. While the effect did not reach significance for the lower skill readers, the same pattern of effects was found for transfer words as trained words. These results support that parietotemporal stimulation affected learning of grapheme-phoneme mappings at the letter level, and did not simply improve route memorization of trained whole word forms. Previous orthographic learning studies have shown that transfer depends on the type of instruction received during training (Bitan et al., 2005; Cao et al., 2013; Mei et al., 2014; Hirshorn et al., 2016; Taylor et al., 2017), even when training is not on individual letters, but on entire word forms (Yoncheva et al., 2010, 2015). Yoncheva et al. (2010) taught participants to read words using the same orthography, but directed attention to either grapheme-phoneme mappings at the letter level or word level. While both groups achieved high accuracy on explicitly trained words, only the group whose attention was directed towards letter-level mappings were able to identify novel words (Yoncheva et al., 2010). In the current study, all participants received the same instructions with explicit attention to the letter-level mappings embedded within the words. Transfer ability was thus not modulated by instruction, but rather by individual differences in pre-training reading skill and parietotemporal stimulation. Therefore, individual differences in skill and neural function prior to training influence learning of grapheme-phoneme mappings which transfers to untrained material.

Despite the skill by stimulation interaction on acquisition rates, parietotemporal stimulation benefitted retention of trained material across all skill levels. This result suggests parietotemporal stimulation may have a differential effect on initial learning and consolidation, and these two stages interact with baseline skill differently. Previous studies examining the effect of stimulation during cognitive training have shown differential effects on initial and later performance (Reis et al., 2009, 2015; Martin et al., 2014), possibly due to a specific effect on consolidation (Alonzoa et al., 2012). In some cases, there are no immediate effects of stimulation, and benefits only emerge after a delay period (Antonenko et al., 2018). Thus, while parietotemporal stimulation interacted with skill to affect acquisition, stimulation may be more universally beneficial to consolidation of learned material. However, the long-term benefits of stimulation were only seen for explicitly trained words and did not transfer to novel words. While transfer effects of tDCS are inconsistent, several studies, including Costanzo et al. (2016b), showed long term benefits of stimulation to tasks that were not performed during the initial training period (for review see Berryhill, 2017). One possible explanation for the lack of maintained transfer effects seen in the current study is the spacing of stimulation sessions. Work examining tDCS enhanced working memory training has shown that stimulation has a greater effect when spaced a few days apart (Au et al., 2016). The majority of stimulation sessions were in the current study were on concurrent days, and no session took place more than 48 h apart. In contrast, the Costanzo et al. (2016b) study delivered three stimulation session over the course of a week. Thus, not only the type of training, but also the timing of stimulation sessions, may be an important factor for determining the optimal design of a tDCS facilitated intervention.

The current study provides promising evidence for parietotemporal stimulation enhancing training on grapheme-phoneme mapping for lower skill readers. Yet, the current study does not allow us to make a definitive statement regarding the specificity of parietotemporal stimulation or the underlying source of these behavioral effects. We chose to stimulate the parietotemporal cortex given its demonstrated role in grapheme-phoneme mapping. However, this area is also associated with cognitive skills such as visual attention, which can also influence reading skill (Bosse et al., 2007; Shaywitz and Shaywitz, 2008; Vidyasagar and Pammer, 2010; Gabrieli and Norton, 2012; Heim et al., 2015). Studies using a similar target site have also shown stimulation can affect visual attention (Minamoto et al., 2014) and working memory (Hill et al., 2016; Trumbo et al., 2016; Möller et al., 2017). These cognitive mechanisms are related to grapheme-phoneme processing, and thus may have mediating roles on the relationship between reading skill, parietotemporal stimulation and grapheme-phoneme mapping. More comprehensive profiles of reading ability may provide additional insights as to the type of reader most likely to respond to stimulation enhanced training. Further, the effects of stimulation can spread to regions functionally and structurally connected to the target region (Turi et al., 2012; Bikson et al., 2013; Park et al., 2013; Choe et al., 2016). It is therefore possible that stimulation additionally affected related reading regions such as the inferior frontal gyrus and occipitotemporal cortex. Conversely, stimulation to any of these connected regions could also potentially result in the same behavioral effects. The spreading effects of stimulation may have acted in conjunction with stimulation to the parietotemporal cortex to affect learning to a greater degree than expected compared to stimulation of parietotemporal cortex in isolation. Given the anatomical and functional connection between parietotemporal and occipitotemporal cortex (Yeatman et al., 2013), parietotemporal cortex stimulation may be more beneficial to reading skill compared to other stimulation targets (Younger et al., 2016). Neuroimaging data could be used to address how neuroanatomy interacts with stimulation to affect behavior.

## Conclusion

The current study provides evidence that the parietotemporal cortex plays an influential role in learning grapheme-phoneme mappings. Parietotemporal stimulation enhanced acquisition of letter-sound mappings of a novel orthography in lower skill readers, and this knowledge was both generalized to untrained material and maintained over a delay period. Thus, parietotemporal stimulation may be an effective tool to support reading instruction for those who struggle by both enhancing existing grapheme-phoneme mappings and supporting the acquisition of new ones. However, stimulation did not benefit all readers equally; higher skill readers were negatively affected, possibly because stimulation interfered with the optimal division of labor between processing pathways. Thus, while parietotemporal function is critical to new learning, its role in continued reading improvement likely changes as readers progress in skill.

## Ethics Statement

This study was carried out in accordance with the recommendations of the Institutional Review Board at the University of Texas at Austin with written informed consent from all subjects. All subjects gave written informed consent in accordance with the Declaration of Helsinki. The protocol was approved by the Institutional Review Board at the University of Texas at Austin.

## Author Contributions

JWY and JRB conceived and designed the experiments; analyzed and interpreted the data. JWY performed the experiments; drafted the manuscript. JRB revised the manuscript. All the authors read and approved the final version of the manuscript.

## Conflict of Interest Statement

The authors declare that the research was conducted in the absence of any commercial or financial relationships that could be construed as a potential conflict of interest.
