# Supplementary material for: Parietotemporal Stimulation Affects Acquisition of Novel Grapheme-Phoneme Mappings in Adult Readers
Source: Front Hum Neurosci. 2018 Mar 23;12:109. doi: 10.3389/fnhum.2018.00109 (PMC5876236; doi:10.3389/fnhum.2018.00109)
Supplement: Supplementary file 1 [file Table_1.DOCX]

# Supplementary Table 1. Artificial orthography stimuli, their pronunciation and their English orthography equivalent.

| **List** | **Artificial Orthography** | **Pronunciation** | **English Orthography** |
| --- | --- | --- | --- |
| Training Set | 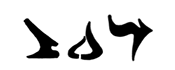 | /baek/ | back |
|  | 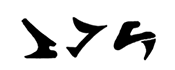 | /bid/ | bead |
|  | 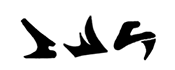 | /bɪd/ | bid |
|  | 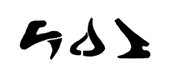 | /daeb/ | dab |
|  | 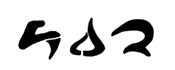 | /daem/ | dam |
|  | 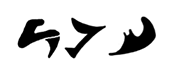 | /dir/ | dear |
|  | 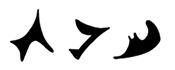 | /gir/ | gear |
|  | 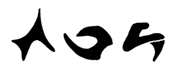 | /gɑd/ | god |
|  | 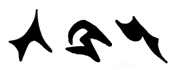 | /gət/ | gut |
|  | 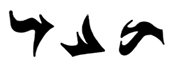 | /kɪn/ | kin |
|  | 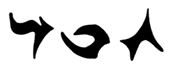 | /kɑg/ | cog |
|  | 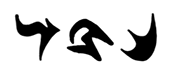 | /kəs/ | cuss |
|  | 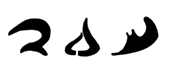 | /maer/ | mar |
|  | 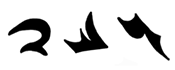 | /mɪt/ | mitt |
|  | 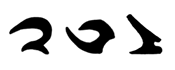 | /mɑb/ | mob |
|  | 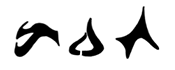 | /naeg/ | nag |
|  | 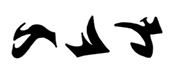 | /nɪp/ | nip |
|  | 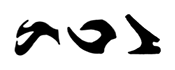 | /nɑb/ | knob |
|  | 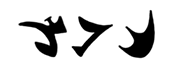 | /pis/ | peace |
|  | 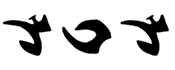 | /pɑp/ | pop |
|  | 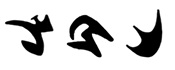 | /pəs/ | pus |
|  | 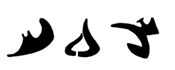 | /raep/ | rap |
|  | 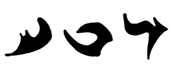 | /rɑk/ | rock |
|  | 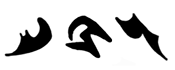 | /rət/ | rut |
|  | 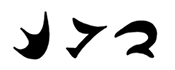 | /sim/ | seam |
|  | 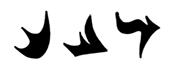 | /sɪk/ | sick |
|  | 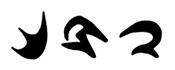 | /səm/ | sum |
|  | 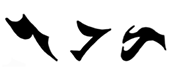 | /tin/ | teen |
|  | 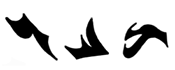 | /tɪn/ | tin |
|  | 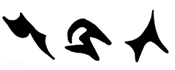 | /təg/ | tug |
| Transfer Set 1 | 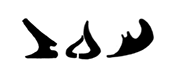 | /baer/ | bar |
|  | 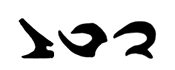 | /bɑm/ | bomb |
|  | 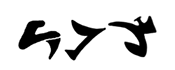 | /dip/ | deep |
|  | 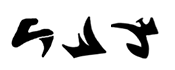 | /dɪp/ | dip |
|  | 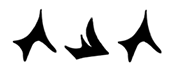 | /gɪg/ | gig |
|  | 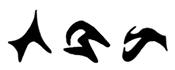 | /gən/ | gun |
|  | 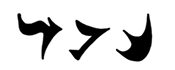 | /kis/ | keys |
|  | 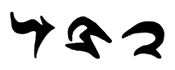 | /kəm/ | come |
|  | 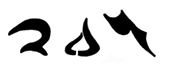 | /maet/ | mat |
|  | 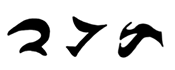 | /min/ | mean |
|  | 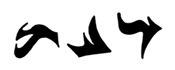 | /nɪk/ | nick |
|  | 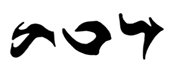 | /nɑk/ | knock |
|  | 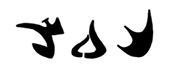 | /paes/ | pass |
|  | 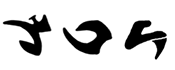 | /pɑd/ | pod |
|  | 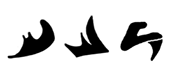 | /rɪd/ | rid |
|  | 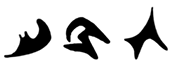 | /rəg/ | rug |
|  | 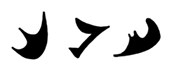 | /sir/ | sear |
|  | 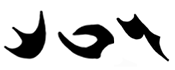 | /sɑt/ | sought |
|  | 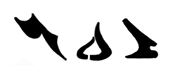 | /taeb/ | tab |
|  | 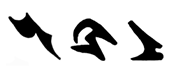 | /təb/ | tub |
| Transfer Set 2 | 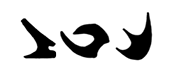 | /bɑs/ | boss |
|  | 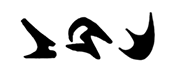 | /bəs/ | bus |
|  | 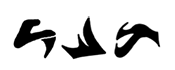 | /dɪn/ | din |
|  | 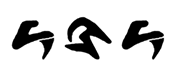 | /dəd/ | dud |
|  | 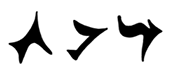 | /gik/ | geek |
|  | 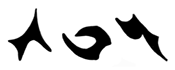 | /gɑt/ | got |
|  | 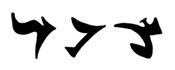 | /kip/ | keep |
|  | 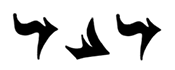 | /kɪk/ | kick |
|  | 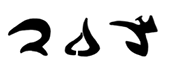 | /maep/ | map |
|  | 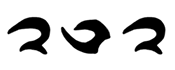 | /mɑm/ | mom |
|  | 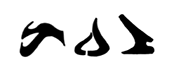 | /naeb/ | nab |
|  | 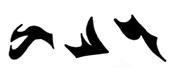 | /nɪt/ | knit |
|  | 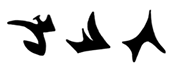 | /pɪg/ | pig |
|  | 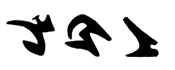 | /pəb/ | pub |
|  | 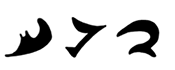 | /rim/ | ream |
|  | 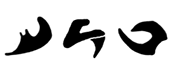 | /rɑd/ | rod |
|  | 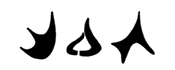 | /saeg/ | sag |
|  | 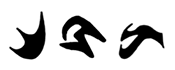 | /sən/ | sun |
|  | 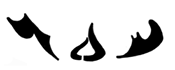 | /taer/ | tar |
|  | 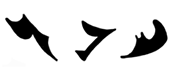 | /tir/ | tear |
| Transfer Set 3 | 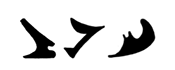 | /bir/ | beer |
|  | 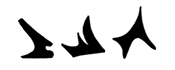 | /bɪg/ | big |
|  | 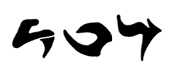 | /dɑk/ | dock |
|  | 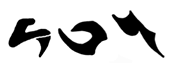 | /dɑt/ | dot |
|  | 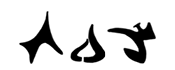 | /gaep/ | gap |
|  | 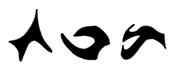 | /gɑn/ | gone |
|  | 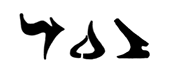 | /kaeb/ | cab |
|  | 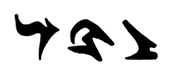 | /kəb/ | cub |
|  | 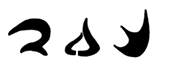 | /maes/ | mass |
|  | 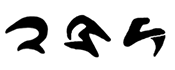 | /məd/ | mud |
|  | 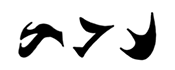 | /nis/ | knees |
|  | 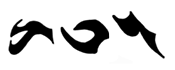 | /nɑt/ | not |
|  | 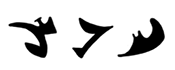 | /pir/ | peer |
|  | 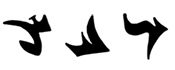 | /pɪk/ | pick |
|  | 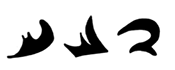 | /rɪm/ | rim |
|  | 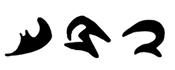 | /rəm/ | rum |
|  | 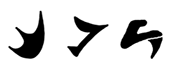 | /sid/ | seed |
|  | 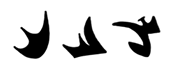 | /sɪp/ | sip |
|  | 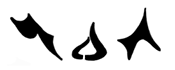 | /taeg/ | tag |
|  | 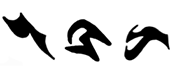 | /tən/ | tun |
| Transfer Set 4 | 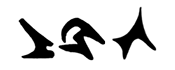 | /bəg/ | bug |
|  | 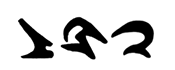 | /bəm/ | bum |
|  | 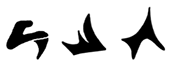 | /dɪg/ | dig |
|  | 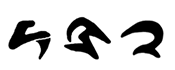 | /dəm/ | dumb |
|  | 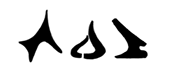 | /gaeb/ | gab |
|  | 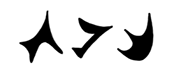 | /gis/ | geese |
|  | 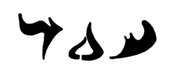 | /kaer/ | car |
|  | 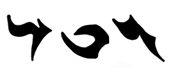 | /kɑt/ | cot |
|  | 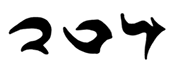 | /mɑk/ | mock |
|  | 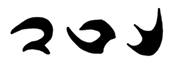 | /mɑs/ | moss |
|  |  | /nir/ | near |
|  |  | /nɑd/ | nod |
|  |  | /pɪt/ | pit |
|  |  | /pən/ | pun |
|  |  | /rik/ | reek |
|  |  | /rɪb/ | rib |
|  |  | /saed/ | sad |
|  |  | /sip/ | seep |
|  |  | /taen/ | tan |
|  |  | /tɪp/ | tip |
| Transfer Set 5 |  | /bim/ | beam |
|  |  | /bɪb/ | bib |
|  |  | /din/ | dean |
|  |  | /dɑg/ | dog |
|  |  | /gaes/ | gas |
|  |  | /gəm/ | gum |
|  |  | /kɪd/ | kid |
|  |  | /kɪs/ | kiss |
|  |  | /mɑp/ | mop |
|  |  | /məg/ | mug |
|  |  | /naet/ | gnat |
|  |  | /nət/ | nut |
|  |  | /paed/ | pad |
|  |  | /paek/ | pack |
|  |  | /rir/ | rear |
|  |  | /rɑb/ | rob |
|  |  | /sin/ | seen |
|  |  | /sɪr/ | sir |
|  |  | /tɑp/ | top |
|  |  | /tək/ | tuck |
